# Nrf2: A Main Responsive Element of the Toxicity Effect Caused by Trichothecene (T-2) Mycotoxin

**DOI:** 10.3390/toxics11040393

**Published:** 2023-04-21

**Authors:** Youshuang Wang, Yu Liu, Tingyu Huang, Yunhe Chen, Wenxi Song, Fengjuan Chen, Yibao Jiang, Cong Zhang, Xu Yang

**Affiliations:** 1 College of Veterinary Medicine, Henan Agricultural University, No. 15 Longzihu University Park, Zhengdong New District, Zhengzhou 450002, China; 2 College of Animal Science and Technology, Henan Agricultural University, Zhengzhou 450002, China

**Keywords:** Nrf2, T-2 toxin, oxidative stress, antioxidation

## Abstract

T-2 toxin, the most toxic type A trichothecene mycotoxin, is produced by Fusarium, and is widely found in contaminated feed and stored grains. T-2 toxin is physicochemically stable and is challenging to eradicate from contaminated feed and cereal, resulting in food contamination that is inescapable and poses a major hazard to both human and animal health, according to the World Health Organization. Oxidative stress is the upstream cause of all pathogenic variables, and is the primary mechanism through which T-2 toxin causes poisoning. Nuclear factor E2-related factor 2 (Nrf2) also plays a crucial part in oxidative stress, iron metabolism and mitochondrial homeostasis. The major ideas and emerging trends in future study are comprehensively discussed in this review, along with research progress and the molecular mechanism of Nrf2’s involvement in the toxicity impact brought on by T-2 toxin. This paper could provide a theoretical foundation for elucidating how Nrf2 reduces oxidative damage caused by T-2 toxin, and a theoretical reference for exploring target drugs to alleviate T-2 toxin toxicity with Nrf2 molecules.

## 1. Introduction

A global issue of food safety is mycotoxin poisoning, which threatens the health of people and animals. A common fusaritoxin generated by several *Fusaria* (*F. poae*, *F. tricinctum* and *F. sporotichioides*) is called T-2 toxin, which is the most common mycotoxin [1]. T-2 toxin is frequently present in foods such as corn, rice, wheat, barley [2], and has also been discovered in water and Chinese herbs [3]. In 188 grain and bedding straw samples collected on Swedish pig farms in 2011 and 2012, T-2 toxin was observed in 29% of the samples [1], and T-2 toxin contamination incidence of up to 79.5% was found in 420 samples of feed from Shandong Province, China [4]. T-2 toxin prevalence grew from 23% in 2018 [5] to 38% in 2020 [1], according to Biomin’s worldwide mycotoxin report, which examined 8721 agricultural products from 75 different nations. According to a report on the occurrence of mycotoxins in feed in China in 2020–2021, three or more mycotoxins were detected in more than 1100 samples, with T-2 toxin detected in over 80% of the samples (Report on the occurrence of mycotoxins in Chinese feedstuffs—2020–2021|PlusVet Animal Health). Moreover, T-2 toxin at a concentration of 0.248–3.61 ng/mL was discovered in the urine of inhabitants of Nanjing Province, China, in 2018 investigations [6]. T-2 toxin is extremely stable and is not vulnerable to the influence of a wide range of environmental conditions, such as heat and ultraviolet radiation [7]. Therefore, it is challenging to completely eliminate T-2 toxin from food and feedstuffs by physicochemical and microbiological methods. Hence, T-2 toxin was identified by the World Health Organization and the Food and Agricultural Organization as the most hazardous natural source when the environment and food are contaminated [8,9].

*Fusarium* contamination can occur when maize, rice, barley, and wheat are cultivated, stored, or processed. *Fusarium* is capable of producing T-2 toxin in environments that are favorable for its survival. The ability of *Fusarium poae* to produce T-2 toxin was strongest in a dark environment with temperatures between 26 °C and 28 °C and a relative humidity of roughly 70%. The highest T-2 toxin production was observed in *Fusarium tricinctum* at 7 °C and 80–100% relative humidity [10]. T-2 toxin is lipophilic. When animals or humans eat grain or processed food infected with *Fusarium*, T-2 toxin enters the body through the gastric mucosa and produces toxic effects. T-2 toxin infection can occur in farmers who are exposed to contaminated hay or grain, or dust produced by the contaminated hay and grain [7,11]. T-2 toxin can cause neurotoxicity, nephrotoxicity, cardiovascular toxicity, reproductive toxicity, immunotoxicity hepatotoxicity and carcinogenic effects after entering the body [12,13].

During World War II, it was discovered that T-2 toxin was the primary pathogenic agent of alimentary toxic aleukia, which is marked by vomiting, diarrhea, leukopenia, nausea and bleeding [14]. According to epidemiological studies, T-2 toxin is most likely to blame for Kashin-Beck disease [15,16], Keshan disease, beriberi-linked cardiac insufficiency [17], Alzheimer’s depression and Parkinson’s disease [18]. Therefore, it is clear that T-2 toxin poses a risk to human and animal health.

## 2. Toxicity Mechanism of T-2 Toxin

**Figure 1:**
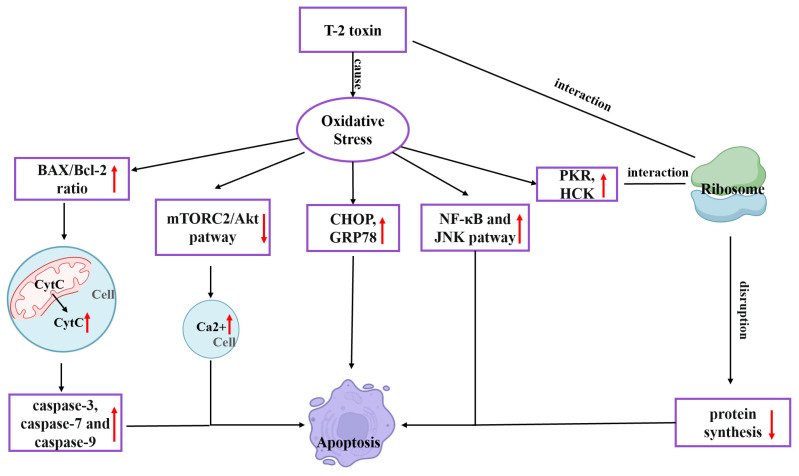
Mechanism of the toxic effects of T-2 toxin.

T-2 toxin undergoes metabolism to form epoxides, which are highly poisonous substances that react with nucleophiles and encourage the creation of reactive oxygen species (ROS). Oxidative stress in the body is further exacerbated by excessive ROS. The 60S ribosomal subunit’s peptidyl transferase can be affected by T-2 toxin, limiting the synthesis of peptide bonds and proteins [19]. Numerous studies have shown that T-2 toxin-generated ROS is the primary cause of harm in broiler and rabbit hepatocytes [20,21], bovine Leydig cells [22], rat ovarian granulosa cells [23], mouse kidneys [24], and mouse PC12 cells [18]. Consequently, it is widely accepted that oxidative stress is the primary reason for T-2 toxin’s toxicity. While several pathogenic changes, such as autophagy, endoplasmic reticulum stress (ERS), mitochondrial damage, inflammatory response and ribosome stress all have oxidative stress as their upstream cause, the final process of these pathological changes is attributed to cell death [25,26]. Increased free Ca^2+^ concentration, DNA methylation, the rapamycin 2 (mTORC2)/Akt pathway, the ERS-related pathway, and the caspase-mediated mitochondrial apoptosis pathway are possible cell-death processes [9]. T-2 toxin causes mitochondrial apoptosis in chicken flesh hepatocytes. By producing ROS, raising the BAX/BCL-2 ratio, and moving the apoptosis pigment C (CytC) from the mitochondria to the cytoplasm, CytC stimulates the activation of caspase-3, caspase-7 and caspase-9 [27]. In mouse TM3 Leydig cells, T-2 toxin can block the mTORC2/Akt pathway by generating ROS, raising the intracellular free Ca^2+^ concentration, and leading to apoptosis [28]. By generating ROS, T-2 toxin can stimulate the expression of RNA-activated protein kinase R (PKR) and hematopoietic cell kinase (HCK). PKR and HCK act on ribosomes to cause ribotoxic stress response, which subsequently induces apoptosis [9]. The NF-κB inflammatory pathway and c-jun N-terminal kinase (JNK) pathway are triggered, and the synthesis of inflammatory mediators (TNF-, IL-1, and IL-6) is greatly elevated in the body by T-2 toxin-caused oxidative stress. At the same time, the expression of ERS indicators CHOP and GRP78 involved in apoptosis increases, followed by apoptosis [29]. T-2 toxin toxicity mechanism is illustrated in Figure 1.

## 3. Physiological Function of Nrf2

**Figure 2:**
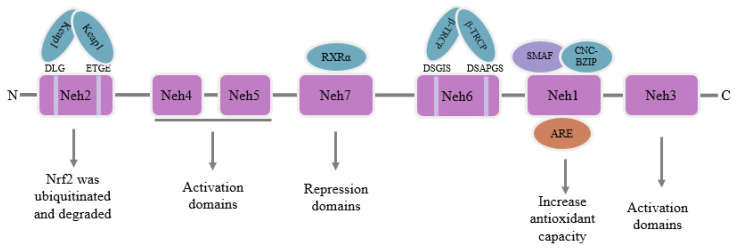
Schematic diagram of the structure of Nrf2.

Nuclear factor E2-related factor 2 (Nrf2), a transcription factor belonging to the Cap’n’Collar (CNC) family [30], includes seven Nrf2-ECH homeodomains (Neh), Neh1-Neh7 [31]. The Neh1 domain is situated at the C-terminal region of Nrf2 and is crucial for regulating Nrf2 activity. Neh1 and small musculoaponeurotic fibrosarcoma proteins (sMAF) can combine to create a dimer through the CNC basic region leucine zipper (CNC-bZIP) structure. The dimer helps Nrf2 recognize and bind to the antioxidant response element (ARE) sequence in nuclear DNA, and then stimulates Nrf2 to initiate the transcription of phase II detoxification enzyme genes or antioxidant enzyme genes to improve the antioxidant capacity of the body [32]. The Neh2 domain is situated at the N-terminal zone of Nrf2, which contains highly conserved sequences of DLG and ETGE and specifically binds to Kelch-like ECH-associated protein 1 (Keap1). After binding, it mediates Nrf2 ubiquitination and degradation in the cytoplasm [33]. The Neh3 domain is also situated at the C-terminal region of Nrf2 and binds to the pigment atpase/helicase DNA binding protein 6 to activate and adjust Nrf2 target gene transcription activity [34]. The Neh4 and Neh5 can cooperate with Neh3 to promote Nrf2 activation [35]. The Neh6 domain mainly contains highly conserved sequences of DSGIS and DSAPGS, which bind to E3 ubiquitin ligase β-transducin repeats-containing proteins (β-TRCP). After binding, the domain mediates the degradation of Nrf2 in oxidative stress cells [36]. Neh7 inhibits the transcription of Nrf2 target genes by interacting with the retinoic X receptor α (RXRα) [37]. A conceptual representation of Nrf2’s structure is depicted in Figure 2.

Nrf2 is a newly discovered signaling pathway that is involved in oxidative stress, iron metabolism, maintenance of mitochondrial function, apoptosis and inflammatory response processes in the body [38]. Under normal conditions, Nrf2 is ubiquitinated and degraded after binding to Keap1, and low levels of Nrf2 content are maintained. As the body experiences stress, is triggered by toxins, and generates an excessive amount of electrophiles, Keap1 and Nrf2 become uncoupled as Nrf2 enters the nucleus and binds to ARE, boosting the expression of its downstream target genes [39], which are involved in:

(1) Antioxidant and detoxification abilities: heme oxygenase 1 (HO-1) [40], NADPH-Quinone oxidoreductase 1 (NQO1) [41], superoxide dismutase (SOD), catalase (CAT), glutathione-S-transferase (GST) [42], malic enzyme (ME1) which maintains NADPH stability [43,44,45], glutamate-cysteine ligase catalytic (GCLC) and glutamate cysteine ligase modifier (GCLM) [46].
(2) Iron metabolism homeostasis: ferritin H (FTH) [47], ferritin L (FTL) [48], cystine/glutamate transporter xCT (SLC7A11), glutathione peroxidase 4 (GPX4) [49] and thioredoxin (Trx) [50].
(3) Autophagy: P62/SQSTM1 [51].

Nrf2 is also associated with other pathways, which are involved in:

(1) Mitochondrial homeostasis: peroxisome proliferator-activated receptor-gamma coactivator-1α (PGC-1α) [52], nuclear respiratory factor 1 (NRF1), nuclear respiratory factor 2, mitochondrial transcription factor A (TFAM) and putative kinase protein 1 (PINK1) [53].
(2) Endoplasmic reticulum homeostasis: activating transcription factor 3 (ATF3) [54].
(3) Apoptosis: caspase molecule and BAX/Bcl-2 [55].
(4) Autophagy: PI3K/Akt signal pathway, mechanistic target of rapamycin (mTOR) [55,56].
(5) Inflammatory response: nuclear factor-κB pathway (NF-κB pathway) [57].

Nrf2 downstream target genes and Nrf2 non-downstream target genes are shown in Table 1.

Nrf2 is engaged in cellular life processes that maintain iron metabolic homeostasis, mitochondrial homeostasis, and oxidative stress homeostasis. Therefore, this article summarizes recent progress in research on the function of Nrf2 molecules in the toxic effects of T-2 toxin. We intend to offer a theoretical guide for reducing T-2 toxin toxicity and comprehending the toxin’s toxicity mechanism in relation to Nrf2.

**Table 1:** Nrf2 downstream target genes and non-downstream target genes.

	Nrf2 Downstream Target Genes	Nrf2 Non-Downstream Target Genes
Antioxidant and detoxification abilities	HO-1 [40], NQO1 [41], SOD, CAT, GST [42], ME1 [43,44,45], GCLC, GCLM [46]	
Iron metabolism Homeostasis	FTH [47], FTL [48], SLC7A11 [41], GPX4 [49], Trx [50]	
Autophagy	P62/SQSTM1 [51]	PI3K/Akt, mTOR [55,56]
Mitochondrial homeostasis		PGC-1α [52], NRF1, TFAM, PINK1 [53]
Endoplasmic reticulum Homeostasis		ATF3 [54]
Apoptosis		caspase molecule, BAX and Bcl-2 [55]
Inflammatory response		NF-κB pathway [57]

## 4. The Role of Nrf2 in Toxic Effects Caused by T-2 Toxin

A summary of all studies of Nrf2 relating to the toxic effect of T-2 toxin induction is in Table 2.

### 4.1. Effect of Nrf2 on Nephrotoxicity Caused by T-2 Toxin

**Figure 3:**
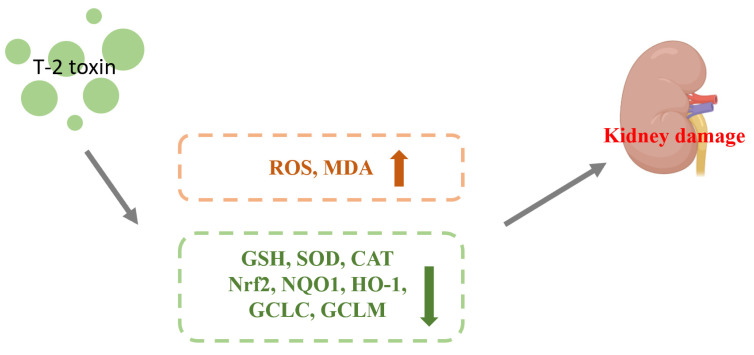
Mechanism of nephrotoxicity induced by T-2 toxin. T-2 toxin causes T-2 toxin leads to increased ROS and MDA contents and decreased expression of GSH, SOD, CAT, Nrf2, NQO1, HO-1, GCLC and GCLM, which leads to kidney damage.

Zhang et al. gavaged mice with 0.5, 1, and 2 mg/kg BW of T-2 toxin for 28 d and found that the levels of ROS and the lipid peroxidation end product malondialdehyde (MDA) were increased, while the level of glutathione (GSH) and SOD and CAT activities were decreased by T-2 toxin in the kidney, resulting in renal oxidative stress in the mice. T-2 toxin increased the expression of the Nrf2 protein and its downstream target genes (NQO1, HO-1, SOD, CAT, GCLC, and GCLM), indicating that the Nrf2 molecule was activated in the kidney. Meanwhile, correlation analysis was used to compare oxidative stress and Nrf2 pathways. The expression of Nrf2 and its downstream target genes in the renal tissues were demonstrated to positively correlate with ROS and MDA contents and negatively correlate with GSH content and SOD and CAT activities, indicating that activated Nrf2 is involved in the oxidative stress caused by T-2 toxin. Results revealed that the kidney may activate Nrf2 and boost the expression index of its downstream target genes (NQO1, HO-1, SOD, CAT, and GCLM) in response to T-2 toxin damage to the kidney. This would counteract the renal oxidative stress brought on by T-2 toxin [26]. The mechanism of T-2 toxin-induced nephrotoxicity is shown in Figure 3.

### 4.2. Effect of Nrf2 on Hepatotoxicity Caused by T-2 Toxin

**Figure 4:**
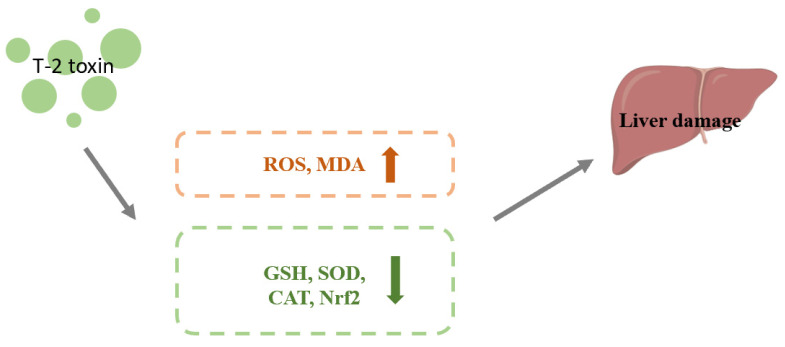
Mechanism of hepatotoxicity induced by T-2 toxin. T-2 toxin leads to increased ROS and MDA contents and decreased expression of GSH, SOD, CAT and Nrf2, which leads to liver damage.

Benjamin et al. conducted an oral administration of 1.82 mg/kg BW T-2 toxin to carp juveniles and removed the liver for analysis after 0, 8, 16, or 24 h. T-2 toxin enhanced the amount of ROS and MDA and decreased GSH content and Nrf2 protein expression. This discovery suggested a possible link between T-2 toxin-induced oxidative stress in the liver of juvenile carp and the suppression of Nrf2 expression. The liver is the largest digestive gland and detoxification organ in the body. The function of Nrf2 in T-2 toxin-induced hepatotoxicity is not entirely understood, so more research is required to shed light on this issue [58]. The mechanism of T-2 toxin-induced hepatotoxicity is shown in Figure 4.

### 4.3. Effect of Nrf2 on Immunotoxicity Caused by T-2 Toxin

The thymus is a key site of development and maturation of T lymphocytes, which play a crucial part in cellular immunity [62], while the spleen is a key site of development and maturation of B lymphocytes, which play a crucial part in humoral immunity [63]. Therefore, the thymus and spleen play a crucial part in protecting the body from pathogens and optimizing immune system function throughout life.

Zhu and Kong et al. gave mice a 4 mg/kg BW intraperitoneal injection of T-2 toxin and harvested the thymus and spleen from the animals 15 h later. Exposure to T-2 toxin led to an excessive buildup of ROS and MDA, as well as a drop in GSH levels, SOD activity, and total antioxidant capacity (T-AOC). Nevertheless, in the thymus and spleen, T-2 toxin significantly boosted Keap1 protein expression while reducing Nrf2 and HO-1 protein expression. This outcome showed that inhibition of Nrf2 and HO-1 protein expression may be associated with the oxidative stress induced by T-2 toxin in the thymus and spleen. The mitogen-activated protein kinase (MAPK) signaling pathway, an important signaling route in cell biology, is affected by oxidative stress. The MAPK family includes JNK, p38 protein kinases (p38), and extracellular regulated protein kinases (ERK). Under conditions of oxidative stress, an excess of ROS activated the apoptosis signal-regulating kinase 1, which in turn activated JNK, p38, and ERK. The production and transcription of genes related to apoptosis were encouraged by these proteins once they were delivered into the nucleus [64]. After T-2 toxin treatment, the ratios of phosphorylated ERK/non-phosphorylated ERK (p-ERK/ERK), phosphorylated p38/non-phosphorylated p38 (p-p38/p38), and phosphorylated ERK/ non-phosphorylated ERK (p-JNK/JNK) rose in the mice thymus and spleen, indicating that the T-2 activated the MAPK signaling pathway [4,38].

**Figure 5:**
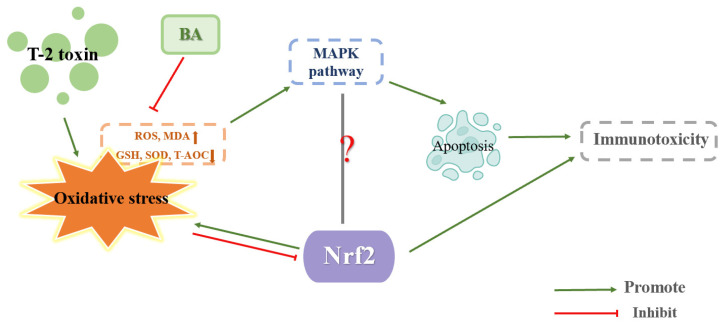
Mechanism of immunotoxicity induced by T-2 toxin.

Betulinic acid (BA), a pentyclic triterpenoid generated from plants, boosts the body’s resistance to oxidative stress, malaria, tumors, inflammation, and HIV while safeguarding cognitive processes [65]. Zhu and Kong et al. treated mice with 0.25, 0.5, and 1 mg/kg BW BA by gavage for 14 d. Then, 9 h after the last gavage of BA, T-2 toxin was intraperitoneally administered to mice at a concentration of 4 mg/kg BW, and the thymus or spleen were taken 15 h after T-2 toxin injection. They discovered that BA reduced MDA and ROS levels and increased SOD and CAT activity, showing that BA reduced the oxidative stress brought on by T-2 toxins in the thymus and spleen. At the same time, the expression of Nrf2 and HO-1 proteins increased as a result of BA decreasing the expression of the protein Keap1, which degrades Nrf2. These findings suggested that BA can decrease T-2 toxin-induced oxidative stress in the thymus or spleen by boosting the expression of Nrf2/HO-1. Furthermore, BA significantly reduced the ratios of p-ERK/ERK, p-p38/p38 and p-JNK/JNK caused by T-2 toxin in the thymus or spleen, proving that BA reduced the MAPK pathway, lessening the toxicity brought on by T-2 toxin. In the present experiment, it appeared that BA alleviated the toxic effects of T-2 toxin by boosting the Nrf2/HO-1 pathway and cutting down the MAPK pathway in the thymus or spleen [4,38]. The mechanism of T-2 toxin-induced immunotoxicity is shown in Figure 5.

BA has the potential to be an effective treatment for T-2 toxin poisoning since it may activate the Nrf2/HO-1 pathway, but its role in other tissues or cells remains to be explored. Meanwhile, how BA activates Nrf2/HO-1 pathway at the molecular level is unclear, and also needs further research and exploration. Is the antagonistic relationship between Nrf2/HO-1 and MAPK in toxicity brought on by T-2 toxin, or is this antagonistic relationship also present in other cell types? If an in vitro experiment can obtain the same results as the in vivo experiment, T-2 toxin could also inhibit the Nrf2/HO-1 pathway and activate the MAPK pathway to aggravate cellular oxidative stress caused by T-2 toxin at the same time the Nrf2/HO-1 pathway was promoted and the MAPK pathway was inhibited by BA, allowing BA to reduce the oxidative stress brought on by T-2 toxin. Consequently, more research is needed.

### 4.4. Effect of Nrf2 on Neurotoxicity Caused by T-2 Toxin

Chaudhary et al. gave mice 1.57 mg/kg BW of subcutaneously delivered T-2 toxin and 5.74 mg/kg BW of percutaneous exposure to T-2 toxin. The mice were put to death 0, 1, 3 and 7 d after exposure. According to these findings, brain ROS content, MDA content, protein carbonyl content, SOD activity and CAT activity were all higher than expected, whereas GSH content was lower, and was more pronounced when administered via the percutaneous route than by subcutaneous injection, indicating that the brain experienced oxidative stress due to T-2 toxin. Meanwhile, Nrf2 and its downstream II detoxification genes (NQO1, GCLC, GCLM and HO-1) were downregulated both in the percutaneous route and subcutaneous injection. The oxidative stress that T-2 toxin inflicted on the brain may have been exacerbated by the potential that it impeded the Nrf2/HO-1 pathway [59].

Pang et al. exposed SH-SY5Y cells for 6 h to T-2 toxin doses of 5 or 10 ng/mL. The ROS level, LDH level and expression of Nrf2 all increased, while glutathione/oxidized glutathione (GSH/GSSG) ratio, expression of factors related to mitochondrial biogenesis (PGC-1α, NRF1 and TFAM), mitochondrial membrane potential (MMP), adenosine triphosphate (ATP) and mitochondrial DNA (mtDNA) copy number decreased, indicating that T-2 toxin caused oxidative stress, apoptosis and mitochondria damage while activating the Nrf2 molecule. Nrf2 acted as a positive regulator of NRF1 by binding with the transcriptional regulating element ARE. NRF1 then activated TFAM and ultimately promoted mitochondrial biogenesis [66]. NRF1 and TFAM are the downstream targets of PGC-1α [67]. A Nrf2 knockdown cell model was created to show how Nrf2 contributes to mitochondrial biogenesis following exposure to T-2 toxin. The results demonstrated that cytotoxicity, ROS production, mitochondrial malfunction, and impairment of mitochondrial biogenesis were higher, while MMP, ATP, mtDNA copy number, and the protein synthesis of PGC-1, NRF1 and TFAM were lower as a result of T-2 toxin after Nrf2 knockdown. These outcomes revealed that T-2 toxin inhibited Nrf2 expression, resulting in oxidative stress, cytotoxicity, and mitochondrial damage [1].

Sun et al. applied T-2 toxin to BV2 cells at concentrations of 1, 2, and 5 ng/mL for 24 h. T-2 toxin enhanced ROS and MDA levels while decreasing CAT and SOD activity, as well as Nrf2 and HO-1 protein expression. ROS are primarily manufactured by mitochondria, and in turn, ROS can also attack the mitochondria, further damaging the mitochondria and forming a vicious cycle. High levels of ROS increased mitochondrial membrane permeability, which led to the breakdown of MMP and mitochondrial malfunction, in turn causing cell death [68]. In the process of apoptosis, the mitochondrial apoptotic pathway is a vital intrinsic path [69]. Increased mitochondrial membrane permeability is regulated by both upregulated BAX and downregulated Bcl-2, after which caspase-3 and caspase-9 are activated by CytC released from mitochondria with increased membrane permeability. The activated caspase-3 could cleave the necessary DNA repair protein poly ADP-ribose polymerase-1 (PARP-1), ultimately promoting apoptosis [3]. The protein release of BAX, cleaved-caspase-3, and cleaved-RARP-1 protein was found to be enhanced as a result of T-2 toxin, while MMP and Bcl-2 protein synthesis were found to be decreased as a result of the toxin, which indicated that T-2 toxin may cause apoptosis of the mitochondrial pathway, oxidative stress and mitochondria damage by inhibiting Nrf2/HO-1 pathway [3].

Zhang et al. administered 10, 20, 40 and 80 ng/mL T-2 toxin to neuroblastoma-2a cells (N2a cells) for 24 h. ROS level and MDA level were upregulated, while GSH content, CAT activity and SOD activity were downregulated, demonstrating that N2a cells suffered oxidative stress as a result of T-2 toxin. The decreased MMP suggested that T-2 toxin damaged the mitochondria. The apoptosis proteins caspase-3, caspase-8, caspase-9, cleaved-PARP-1, pro-apoptotic protein BAX and p53 increased and the anti-apoptotic protein Bcl-XL decreased. It is well known that the p53 plays a part in preserving genomic stability and terminating the cell cycle. The p53 facilitates the association of the death receptor and mitochondria by influencing the synthesis of caspase-8. In the state of oxidative stress, activated p53 can lead to mitochondrial dysfunction or directly activate the expression of BAX, increasing mitochondrial permeability and in turn triggering cell apoptosis [70]. The results indicated that T-2 toxin triggered cell death via the mitochondrial pathway while inhibiting the Nrf2/HO-1 pathway. Nrf2 is involved in apoptosis, mitochondrial biogenesis and other processes. These preliminary results demonstrated that T-2 toxin inhibits the production of Nrf2, and that HO-1 may be strongly related to the oxidative stress, mitochondrial damage, and mitochondrial pathway apoptosis caused by T-2 toxin. After expression of Nrf2 was inhibited, cell activity was lower and apoptosis protein expression of caspase-3 and caspase-9 was more significant, suggesting that the cytotoxicity caused by T-2 toxin was further exacerbated. It appears that blocking Nrf2 may further impair N2a cells’ capacity to launch an antioxidant defense, escalating the oxidative stress brought on by T-2 toxin. This suggests the possibility of lessening the detrimental impact of T-2 toxin by activating Nrf2 [60].

Pei et al. applied T-2 toxin at concentrations of 0.75, 1, 3, 6, or 12 ng/mL to PC12 cells for 24 h. The T-2 toxin generated oxidative stress in cells, as evidenced by the rise in ROS and MDA levels, as well as the decrease in SOD activity, CAT activity and GSH level. T-2 toxin-induced apoptosis was demonstrated by increasing the release of CytC, cleaved caspase-3, caspase-9 and BAX protein. The inflammatory mediators (TNF-α, IL-6, IL-8 and IL-1β) were increased, showing that inflammation was brought on by the T-2 toxin. Here, in a slight departure from prior analysis that found Nrf2 and HO-1 expression to be upregulated when T-2 toxin concentrations were below 6 ng/mL, Nrf2 and HO-1 expression was shown to be downregulated when T-2 toxin concentrations were greater than 6 ng/mL. The temporarily increased expression of Nrf2 and HO-1 may be a cellular self-protective mechanism at work. The decreased Nrf2 and HO-1 expression may be due to the cytotoxicity caused by T-2 toxin exceeding the maximum threshold of the cell self-protection mechanism. It appears that the maintenance of REDOX balance in N2a cells is a dynamic process in which the T-2 toxin reaches the cellular antioxidation tipping point at 6 ng/mL. This result suggests that Nrf2 expression may be based on the T-2 toxin dosage [18].

**Figure 6:**
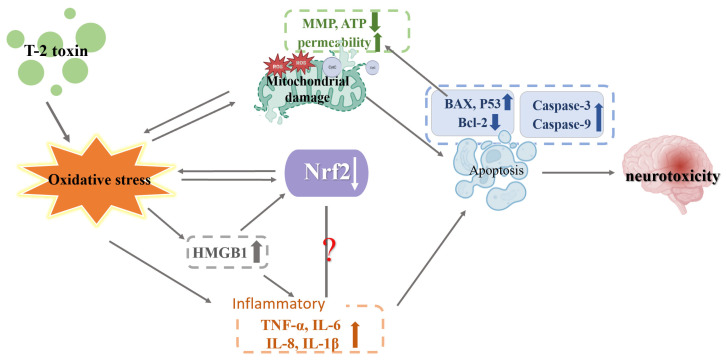
Mechanism of neurotoxicity induced by T-2 toxin. T-2 toxins cause oxidative stress. Oxidative stress leads to mitochondrial damage (decreased MMP and ATP content, increased mitochondrial permeability, ROS production and increased cytochrome exudation), and mitochondrial damage leads to apoptosis (increased expression of caspase-3, caspase-9, BAX and P53, decreased expression of Bcl-2), which results in neurotoxicity. Increased expression of BAX and P53 exacerbates mitochondrial damage, and ROS produced by mitochondrial damage further increases oxidative stress. Oxidative stress leads to decreased Nrf2 expression, which in turn exacerbates oxidative stress. Oxidative stress leads to increased expression of HMGB1, which in turn leads to decreased expression of Nrf2 and increased expression of inflammatory factors (TNF-α, IL-6, IL-8 and IL-1β), and increased expression of inflammatory factors promotes cell apoptosis. Oxidative stress leads to increased expression of inflammatory factors. The relationship between Nrf2 and inflammation is unclear.

High Mobility Group Box 1 (HMGB1), a cellular protein that stimulates and controls inflammatory reaction, has been indicated as a biomarker and potential new therapeutic target for a variety of illnesses [71]. HMGB1 causes the release of inflammatory factors (TNF-α, IL-1β and IL-6). These inflammatory factors may also stimulate HMGB1, creating a positive feedback cycle that strengthens the inflammatory effect [72]. The amount of HMGB1 both inside and outside the PC12 cells rose when Pei et al. exposed them to T-2 toxin at a dosage of 3 ng/mL for 24 h. When the expression of HMGB1 was inhibited, apoptosis, oxidative stress and inflammation in PC12 cells induced by T-2 toxin were alleviated. However, the T-2 toxin’s induction of increased Nrf2 expression was inhibited by HMGB1 expression. HMGB1 may be a signaling molecule that regulates Nrf2 expression; therefore, HMGB1 is promising as a new target for the treatment of T-2 toxin toxicity by mediating Nrf2 expression [18]. The mechanism of T-2 toxin-induced neurotoxicity is shown in Figure 6.

Taken together, in the in vivo experiment, Nrf2 expression was decreased, while SOD and CAT expression, whose transcriptional activity is controlled by Nrf2 [14], were increased, which is worth further investigation. On one hand, the reduction of Nrf2 expression may be due to the dose of T-2 toxin exceeding the brain’s own antioxidant stress threshold. Alternatively, other routes or variables may also modulate the expression of SOD and CAT. In the vitro experiment, whether T-2 toxin causes mitochondrial damage, inflammation and apoptosis is related to the decrease in the Nrf2 molecule as a result of T-2 toxin, so the relationship between the Nrf2 molecule and mitochondrial damage, inflammation and apoptosis must be examined. Furthermore, a new molecule (HMGB1) was discovered to take part in T-2 toxin-induced toxicity. The molecular mechanism between HMGB1 and Nrf2, and whether HMGB1 plays the same role in other cell types and tissues, must also be examined. All of these questions require further exploration.

### 4.5. Effect of Nrf2 on Endocrine Toxicity Caused by T-2 Toxin

Chen et al. administered 5 ng/mL of T-2 toxin to Michigan Cancer Foundation-7 cells (MCF-7 cells) for 24 h. ROS synthesis was elevated, while nitric oxide synthase, CAT, GST, SOD, and the gene and protein expression of the Nrf2 were all diminished. These findings showed that the T-2 toxin caused oxidative stress and reduced the gene and protein expression of the Nrf2. It is noteworthy that increasing Nrf2 expression stopped ROS buildup, demonstrating that the T-2 toxin caused oxidative stress by lowering Nrf2 expression in MCF-7 cells. Activating transcription factor 3 (ATF3), a biological stress gene, is activated when cells respond to stress signals such as adipokines, disease, hypoxia, mycotoxins cytokines and chemokines [73]. Inflammation, ERS, apoptosis oxidative stress, cell cycle arrest and disease can all be brought on by the overexpression of ATF3 [74]. ATF3 expression was elevated by T-2 toxin in the MCF-7 cells. Interestingly, ATF3 knock-down dramatically reduced the production of ROS and increased the expression of Nrf2 in MCF-7 cells exposed to T-2 toxin. Conversely, the expression of Nrf2 was downregulated under ATF3 overexpression. T-2 toxin upregulates AFT3, and the upregulated ATF3 reduces Nrf2 expression, which may be one of the factors contributing to the oxidative stress that T-2 toxin induces. There seems to be an antagonistic relationship between ATF3 and Nrf2. The expression of Nrf2 was increased by inhibiting the expression of ATF3, thereby alleviating the oxidative stress as a result for T-2 toxin. This offers fresh insight into the regulation function of Nrf2 under oxidative stress brought on by the T-2 toxin [61].

**Figure 7:**
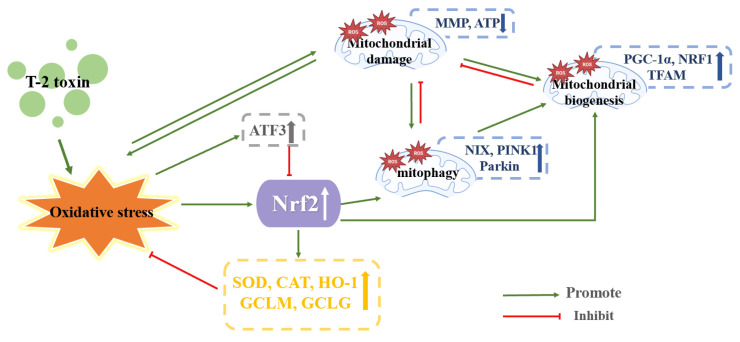
Mechanism of endocrine toxicity induced by T-2 toxin. Blue, yellow, gray, and white arrows, arrows up indicate increased expression and arrows down indicate decreased expression.

Huang et al. treated mice GH3 cells with 10 and 40 nM T-2 toxin for 24 h. The T-2 toxin triggered oxidative stress by increasing intracellular ROS generation and GSH consumption. It is interesting to note that higher levels of SOD, CAT, mitochondrial uncoupling protein and Nrf2 were observed, and GH3 cells appeared to exhibit stronger antioxidant capacity, which may be related to the activation of Nrf2 expression by the T-2 toxin. MMP was decreased, and the enzyme activity of mitochondrial complex I, ATP and marker of oxidative DNA damage 8-OHDG level and the factors participating in mitochondrial biogenesis PGC-1α, NRF1 and TFAM were increased, indicated that T-2 toxin could harm DNA and impair mitochondrial function, but that it could also withstand mitochondrial damage by temporarily boosting mitochondrial biogenesis and function. This may be connected to the increased expression of Nrf2. One of the main generators of intracellular ROS are mitochondria, which are also the primary target of ROS assault [75]. Therefore, impaired mitophagy has positive significance for oxidative stress injury. The proportions of the LC II/LC I, autophagy-related 3 and mitophagy-specific proteins (NIX, PINK1 and Parkin) were enhanced by T-2 toxin. These discoveries indicated that T-2 toxin induced mitophagy. Nrf2 plays a role in regulating PINK1-mediated mitophagy [76]. When using siRNA interference technology to downregulate Nrf2 expression, PINK1 expression was decreased, demonstrating that Nrf2 knockdown can significantly reduce PINK1 expression, reduce damaged mitophagy and aggravate mitochondrial damage. PKA, a crucial kinase in mitochondrial biogenesis, may repair mitochondrial abnormalities brought on by PINK1 depletion [14]. Thus, blockade of PKA with the PKA inhibitor H89 was found to reduce Nrf2 and PINK1 expression, showing that PKA/Nrf2/PINK1/Parkin is a key pathway for regulating mitophagy in GH3 cells by T-2 toxin treatment. Nrf2 may antagonize T-2 toxin-induced apoptosis in GH3 cells by activating mitophagy, which provides some new ideas for reducing the toxicity of T-2 toxin by improving mitochondrial function [14]. It was discovered that the PKA/Nrf2/PINK1/Parkin pathway and the ATF3 molecule were key players in the toxicity caused by the T-2 toxin. T-2 toxin may inhibit the expression of Nrf2 by upregulating the expression of ATF3, suggesting that not only does T-2 toxin directly regulate and control Nrf2 by producing nucleophilic epoxides, but also that ATF3 and Nrf2 interaction’s prospective molecular mechanisms are still mostly understood. Moreover, the PKA/Nrf2/PINK1/Parkin pathway alleviated T-2 toxin-induced toxicity by activating mitophagy, which suggests that we can alleviate T-2 toxin-induced toxicity by increasing mitophagy. However, excessive autophagy can also have toxic effects on cells, so it is vital to determine the threshold of mitophagy. Ultimately, these ideas need to be further explored. The mechanism of T-2 toxin-induced endocrine toxicity is shown in Figure 7.

## 5. Conclusions

**Figure 8:**
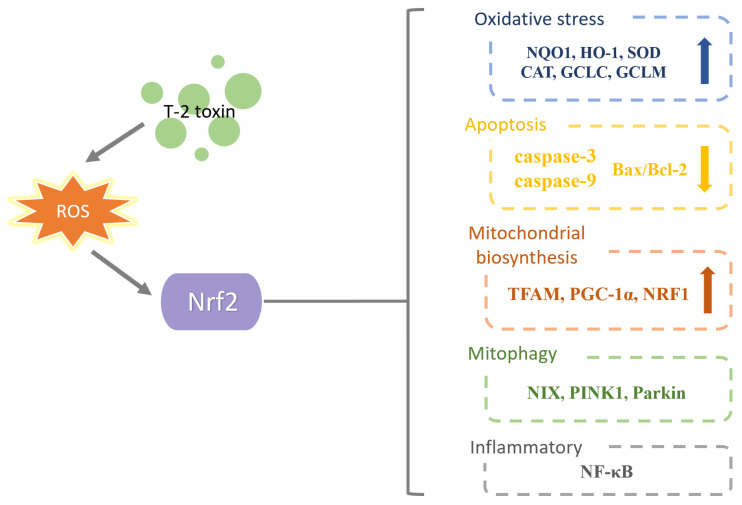
A current line of research on Nrf2 in T-2 toxin toxicity. T-2 toxin causes oxidative stress which affects Nrf2 expression. Nrf2 can increase anti-oxidative stress indicators, reduce apoptosis indicators, increase mitochondrial biogenesis indicators, affect autophagy indicators and affect inflammatory indicators.

The literature mentioned above showed that the T-2 toxin causes oxidative stress, inflammation, apoptosis and mitochondrial damage by affecting the expression of Nrf2. Furthermore, several new Nrf2-related molecules or pathways (HMGB1, ATF3, the Nrf2/HO-1 pathway and the PKA/Nrf2/PINK1/Parkin pathway) were found to take part in the action of the T-2 toxin. The T-2 toxin has a dual effect on Nrf2 expression, which may depend on the dose and duration of T-2 toxin treatment and the animal species or organ. It is still unknown how HMGB1, ATF3, the Nrf2/HO-1 pathway and the PKA/Nrf2/PINK1/Parkin pathway interact with the Nrf2 molecule. In addition to regulating oxidative stress, Nrf2 controls iron metabolism, autophagy, inflammation and mitochondrial homeostasis, and more research is required on these topics. It should also be determined whether Nrf2 can be used as a candidate molecule in the future to lessen or neutralize the harmful effects brought on by T-2 toxin. A current line of research on Nrf2 in T-2 toxin toxicity is exhibited in Figure 8.

## Figures and Tables

**Table 2:** A summary of all investigations on Nrf2 and the harmful effects of T-2 toxin induction.

	Experiment Model	Treatment Mode and Time	Results	Intervention Experiment	Reference
nephrotoxicity	Mice (kidney)	0.5, 1, and 2 mg/kg BW of T-2 toxin gavage for 28 d	The levels of ROS, lipid peroxidation end products malondialdehyde (MDA), the Nrf2 protein and its downstream target genes (NQO1, HO-1, SOD, CAT, GCLC, and GCLM) were increased. The levels of glutathione (GSH), and SOD and CAT’s activities were decreased.		[26]
hepatotoxicity	carp juveniles (liver)	1.82 mg/kg BW T-2 toxin gavage for 8, 16 or 24 h	The amount of ROS and MDA were increased. GSH contents and Nrf2 protein expression were decreased.		[58]
immunotoxicity	Mice (thymus or spleen)	4 mg/kg BW T-2 toxin intraperitoneal injection for 15 h	ROS level, MDA level, Keap1 protein expression and MAPK pathway were increased. GSH levels, SOD activity, T-AOC activity and Nrf2/HO-1 protein expression were decreased.	BA decreased MDA level, ROS level and MAPK pathway. BA increased SOD activity, CAT activity and Nrf2/HO-1 protein expression.	[4,38]
neurotoxicity	Mice (brain)	1.57 mg/kg BW T-2 toxin Subcutaneous injection for 1, 3 and 7 d 5.74 mg/kg BW T-2 toxin Percutaneous exposure for 1, 3 and 7 d	ROS content, MDA content, protein carbonyl content, SOD activity and CAT activity were increased. GSH content, Nrf2 and its downstream II detoxification genes (NQO1, GCLC, GCLM and HO-1) were decreased.		[59]
	SH-SY5Y cells	5 or 10 ng/mL T-2 toxin for 6 h	The ROS level, LDH level and expression of Nrf2 were increased. GSH/GSSG ratio, PGC-1α, NRF1, TFAM, MMP, ATP and mtDNA copy number were deceased.	After Nrf2 knockdown, cytotoxicity, ROS production, mitochondrial malfunction, and impairment of mitochondrial biogenesis were increased. After Nrf2 knockdown, MMP, ATP, mtDNA copy number, PGC-1, NRF1 and TFAM were decreased.	[1]
	BV2 cells	1, 2, and 5 ng/mL T-2 toxin for 24 h	ROS level, MDA level, BAX, cleaved-caspase-3 and cleaved-RARP-1 were increased. CAT activity, SOD activity, Nrf2/ HO-1 protein, MMP and Bcl-2 were decreased.		[3]
	N2a cells	10, 20, 40 or 80 ng/mL T-2 toxin for 24 h	ROS level, MDA level caspase-3, caspase-8, caspase-9, cleaved-PARP-1, pro-apoptotic protein BAX and p53 were increased. GSH content, CAT activity, SOD activity, MMP, Bcl-XL and Nrf2/HO-1 were decreased.	After inhibiting the expression of Nrf2, the cell activity was decreased. After inhibiting the expression of Nrf2, caspase-3 and caspase-9 were increased.	[60]
	PC12 cells	0.75, 1, 3, 6 or 12 ng/mL T-2 toxin for 24 h	ROS level, MDA level, CytC, cleaved caspase-3, caspase-9, BAX and HMGB1 were increased. SOD activity, CAT activity and GSH level were decreased. Nrf2/HO-1 was increased at T-2 toxin < 6 ng/mL, was decreased at T-2 toxin > 6 ng/mL.	After inhibiting HMGB1, apoptosis, oxidative stress and inflammation were decreased. After inhibiting HMGB1, Nrf2 was increased.	[18]
Endocrine toxicity	MCF-7 cells	5 ng/mL T-2 toxin for 24 h	ROS synthesis, ATF3 were increased. Nitric oxide synthase, CAT, GST, SOD, Nrf2 were decreased	After overexpressing Nrf2, ROS synthesis was decreased. After inhibiting ATF3, ROS level was decreased. After inhibiting ATF3, Nrf2 was increased. After overexpressing ATF3, Nrf2 was decreased.	[61]
	GH3 cells	10 and 40 nM T-2 toxin for 24 h	ROS level, GSH-Px, SOD, CAT, UCP, Nrf2, mitochondrial complex I, ATP, 8-OHDG level, PGC-1α, NRF1, TFAM, LC II/LC I, autophagy-related 3, NIX, PINK1 and Parkin were increased. GSH level, MMP were decreased.	After inhibiting Nrf2, PINK1 expression was decreased. After inhibiting PKA, Nrf2 and PINK1 were decreased.	[14]

## Data Availability

Not applicable.
